# Rapid selection of high-affinity DARPins using click display

**DOI:** 10.1093/protein/gzag028

**Published:** 2026-10-03

**Authors:** Joshua D Bryant, Yu Zeng, Monisha Yerram, Karuppiah Chockalingam, Michael Woolley, Raghav Sehgal, Vikas Chonira, Zhilei Chen

**Affiliations:** Department of Microbial Pathogenesis and Immunology, Texas A&M University Health Science Center, 8447 John Sharp Pkwy, Bryan, TX 77807, United States; Department of Microbial Pathogenesis and Immunology, Texas A&M University Health Science Center, 8447 John Sharp Pkwy, Bryan, TX 77807, United States; Department of Microbial Pathogenesis and Immunology, Texas A&M University Health Science Center, 8447 John Sharp Pkwy, Bryan, TX 77807, United States; Department of Microbial Pathogenesis and Immunology, Texas A&M University Health Science Center, 8447 John Sharp Pkwy, Bryan, TX 77807, United States; Department of Microbial Pathogenesis and Immunology, Texas A&M University Health Science Center, 8447 John Sharp Pkwy, Bryan, TX 77807, United States; Department of Microbial Pathogenesis and Immunology, Texas A&M University Health Science Center, 8447 John Sharp Pkwy, Bryan, TX 77807, United States; Department of Microbial Pathogenesis and Immunology, Texas A&M University Health Science Center, 8447 John Sharp Pkwy, Bryan, TX 77807, United States; Xilio Therapeutics, Inc., Waltham, Massachusetts 02451, USA; Department of Microbial Pathogenesis and Immunology, Texas A&M University Health Science Center, 8447 John Sharp Pkwy, Bryan, TX 77807, United States; Department of Biochemistry and Biophysics, Texas A&M University, 300 Olsen Blvd, College Station, TX 77843, United States; Department of Genetics and Genomics, 300 Olsen Blvd. College Station, TX 77843-2128, United States

## Abstract

Rapid and scalable protein binder discovery platforms are essential for accelerating therapeutic and diagnostic development. Click display is a cell-free protein display technology that covalently links nascent proteins to their encoding cDNA in a one-pot reaction within 2 h, enabling rapid *in vitro* selection. Although previously demonstrated to support directed evolution, enrichment efficiency during biopanning was slower than conventional phage display. Here, we report two modifications that significantly enhance click display selection efficiency: (i) selective elution of target-bound complexes using a cleavable biotin linker, and (ii) competitive displacement of non-specific bead binders using a biotinylated blocking protein. These improvements dramatically reduce amplification of non-specific binders and enable efficient enrichment of target-specific binders within three to four rounds of panning. Using a naïve DARPin library, we rapidly enriched binders to interleukin-10 (IL-10) and transforming growth factor-β1 (TGF-β1), identifying clones with low nanomolar binding affinities and receptor-blocking activity. We further applied the optimized protocol to affinity maturation of a PD-L1-binding DARPin, achieving >400-fold improved binding affinity. The best candidate, 1D10, exhibits sub-nanomolar PD-1 inhibition in competition ELISA. These results demonstrate that the optimized click display workflow enables rapid enrichment and affinity maturation of high-affinity DARPins across diverse targets. The platform provides a streamlined, transformation-free approach for accelerating protein engineering and directed evolution.

## Introduction

The ability to engineer high-quality protein binders within days instead of months addresses a societal need for agility in medicine and public health. Pandemic-scale pathogens, fast-evolving cancer neoantigens, and newly discovered biomarkers all demand countermeasures that keep pace with biology’s own speed. The availability of reliable ‘rapid-engineering’ platforms should compress the hit-to-lead cycle so diagnostics can be updated in step with viral variants, neutralizing antibodies can be stockpiled before an outbreak crests, and personalized therapeutics can be tailored to an individual’s tumor in clinically relevant time frames. By cutting development timelines and costs, these platforms should also expand healthcare access by enabling smaller biotech firms and low-resource health laboratories to pursue niche or emerging targets, leading to earlier intervention and more diverse treatment options for patients.

Protein engineering spans a spectrum from human-guided modifications to fully autonomous design loops. Apart from rational design, which entails the screening of a very small number of variants, most current protein engineering methods require protein display – the linking of phenotype (the measurable properties of a protein) and genotype (the DNA/RNA sequence that encodes that variant) – to enable high-throughput selection and quantitative fitness-landscape mapping at massive scale (Doi and Yanagawa 2001, Leemhuis *et al*. 2005). Protein display methods fall into two broad categories: cell-based display and cell-free display.

Cell-based display technologies express the library variants as a fusion to a host coat/surface protein. Phage display tethers peptides or small proteins to pIII or pVIII coat protein on M13 virions, enabling low-cost selection of up to 10^11^ variants (Clackson *et al*. 1991). Yeast-surface display uses Aga2/Aga1 coat protein as the anchor for library variants and sacrifices orders of magnitude in library size (10^7^–10^8^) but gains in its ability to support quantitative flow-cytometric read-outs and disulfide / glycosylation capacity (Boder and Wittrup 2000, Shusta *et al*. 2000, Chao *et al*. 2006). Bacterial display anchors library variants to select *E. coli* surface protein and supports a similar library complexity to phage display (Rutherford and Mourez 2006, Daugherty 2007). Mammalian-cell display delivers human-like protein folding and post-translational modification critical for many therapeutic receptors and antibodies but also suffers from a much smaller library size (10^6^–10^8^) (Ho *et al*. 2006, Zhou *et al*. 2010, Bowers *et al*. 2011). Each of these cell-based display methods is limited by the need for cell transformation, which constrains the library size for all cell-displayed technologies to 10^6^–10^11^. Additionally, because each round of selection or enrichment requires the recovery of library variants through cell growth, cell-based display technologies generally exhibit slow turnaround times (ranging from days/weeks for phage display to weeks/months for mammalian cell display). Typically, improved protein folding quality is associated with smaller effective library sizes and longer durations for each enrichment round.

Cell-free display methods overcome the transformation constraint inherent in cell-dependent selection methods by linking the protein directly to its coding genetic material, negating the need for transformation and greatly expanding the usable library size. mRNA display covalently links the nascent protein to its mRNA *via* a puromycin adaptor (Roberts and Szostak 1997) and allows harsh washes and ultra-large libraries (10^12^–10^14^)(Newton *et al*. 2020). However, since mRNA is unstable, strict RNase-free conditions are necessary to prevent RNA degradation both during puromycin conjugation and library panning. Ribosome display uses a stop codon-free mRNA to stall the ribosome, which tethers the mRNA to the nascent protein. Although significantly simpler than mRNA display (Schaffitzel *et al*. 1999, Lipovsek and Pluckthun 2004, Zahnd *et al*. 2007, Dreier and Plückthun 2011, 2012a, 2018, Plückthun 2012), ribosomal display also requires strict RNase-free conditions during panning. CIS display utilizes a DNA replication initiator protein (RepA) to bind the template DNA (Odegrip *et al*. 2004), enabling panning to proceed in regular buffer without concerns for RNA integrity. However, since RepA is relatively large (33 kDa) and must be fused to the N-terminus of every library member, CIS display is most suited for small proteins and peptides whose N-terminus is not critical to protein folding or function (Mathonet *et al*. 2011). The cDNA display method uses a branched DNA oligo complementary to the mRNA, which both primes cDNA synthesis and enables conjugation to the nascent protein *via* puromycin (Ueno *et al*. 2012), and potentially marries the simplicity of ribosome-display with the stability of the covalent linkage used in mRNA display. However, the branched oligo requires in-house synthesis and suffers from low synthesis yield (<0.5% to 4%) (Naimuddin and Kubo 2016), hampering its broad application.

Previously, we developed a cell-free display method termed ‘click display’ that is potentially advantageous to all current cell-free protein display technologies (Zeng *et al*. 2023). In click display, a branched DNA oligo (named ML), analogous to that used in cDNA display, is generated by a facile click chemistry reaction and is used to covalently link the nascent protein to the coding cDNA. Importantly, all reagents necessary for click display are commercially available, and the entire process from input DNA template to covalently conjugated protein-cDNA complex takes <2 h in a one-pot reaction. Unlike the branched oligo used in cDNA display, which suffers from low synthesis yield and requires extensive purification, ML is produced from commercially available oligos *via* click chemistry with >90% reaction efficiency and is used as is without further purification.

Here, we describe an improved click display library enrichment protocol and its use in the enrichment of designed ankyrin repeat protein (DARPin) pools to three distinct target proteins that have been identified for their potential in modulating the tumor microenvironment. We achieve binder enrichment to IL-10 and TGFβ from a naïve library within one week and rapid affinity maturation of a PD-L1-specific DARPin.

## Results

### Target protein preparation and optimized selection protocol

We describe here an update to the click display panning procedure that we have reported previously (Zeng *et al*. 2023). Biopanning is an iterative selection method in which a diverse protein-display library is exposed to a target, non-binding variants are washed away, and variants bound to the target are immobilized on a solid support and recovered/amplified to enrich for high-affinity, target-specific binders. In click display, the displayed proteins are covalently linked to their coding cDNA (Fig. 1A) and target-bound variants are enriched/amplified *via* PCR after pull-down. In the original click display panning protocol, we used magnetic streptavidin beads to capture a biotinylated target protein, then PCR-amplified the cDNA from the beads directly (Fig. 1B). While this approach has the advantage of expedience, it also amplifies any of the protein-cDNA complexes that non-specifically bind the beads. We found that, depending on the specific target, non-specific binders to the beads can significantly slow down the enrichment process, increasing the challenge of enriching target-specific binders. Although negative selection, in which the library pool is first incubated with empty beads, has been routinely employed by us and others during panning to reduce the prevalence of non-specific binders, we have found negative selection alone to be insufficient in the case of click display (data not shown). In our hands, phage panning typically takes 3–4 rounds to saturate enrichment of target-binding DARPins (Peng *et al*. 2019, Simeon *et al*. 2019, Chonira *et al*. 2023). However, it took 6 rounds to significantly enrich DARPin binders to the model protein Clumping Factor A (ClfA) using click display (Zeng *et al*. 2023), suggesting that the presence of non-specific binders may hamper the panning efficiency.

**Figure 1:**
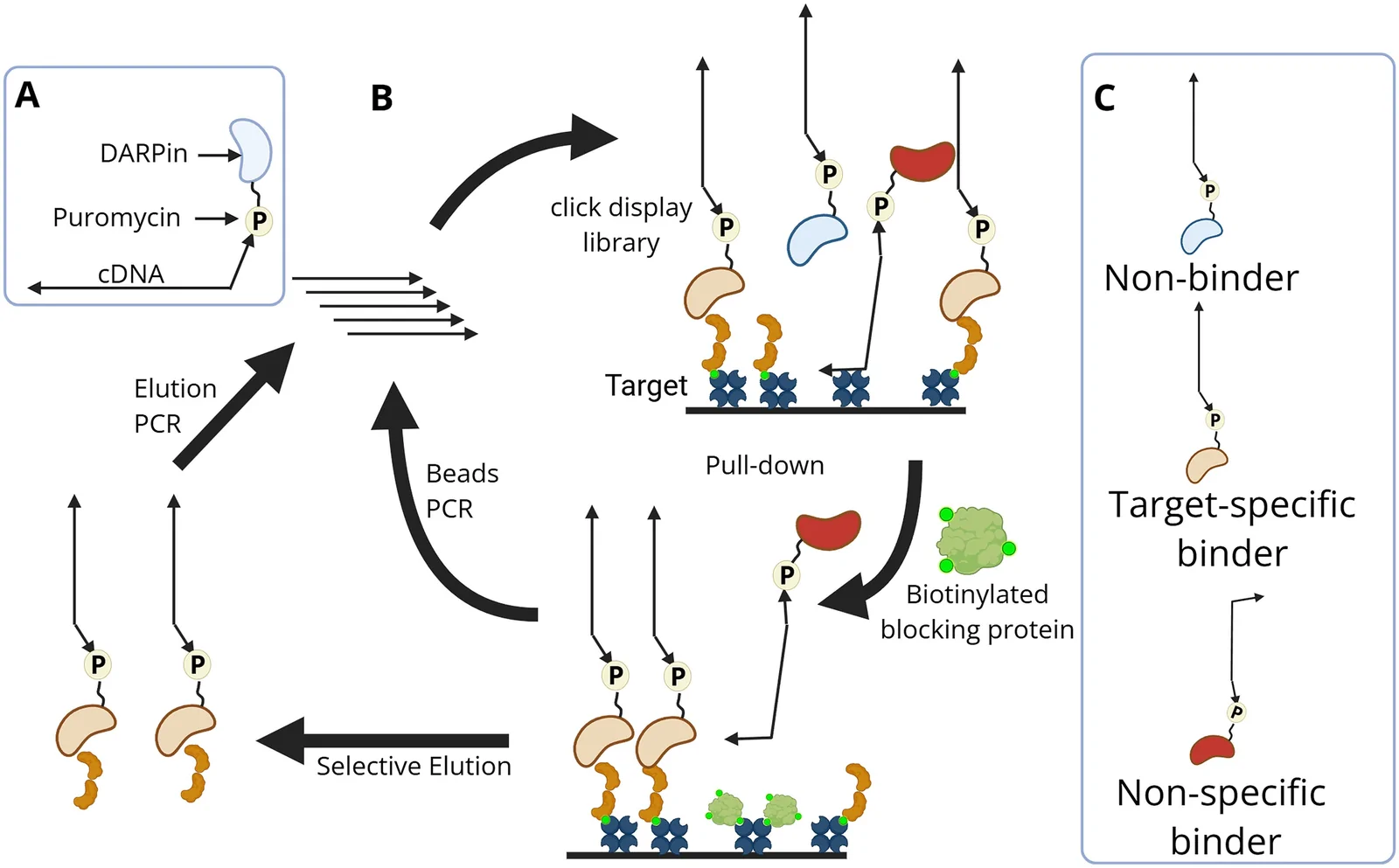
**Overview of click display biopanning. (A)** Schematic of click-displayed protein-cDNA complex. **(B)** Overview of click display biopanning procedure. Both target-specific and non-specific binders are associated with the streptavidin beads and can be amplified *via* bead-PCR. A selective elution procedure that elutes only target-specific binders from the beads should significantly improve the biopanning efficiency. **(C)** Schematic of the three types of protein-cDNA complexes in a click display candidate library. Images were generated using BioRender.

In a click-displayed library, there are three types of protein-cDNA complexes (Fig. 1C): **i)** complexes that bind the target specifically, **ii)** complexes that do not bind the target (non-binders) and **iii)** complexes that bind the solid support non-specifically. In addition to protein molecules that can bind the solid support *via* non-specific charge-based or hydrophobic interactions, non-specific interactions with the solid support can also be mediated *via* cDNA/RNA molecules due to the strong negative charge of the phosphate backbone (Cotten *et al*. 2012, Valencia *et al*. 2013, Reyes *et al*. 2021). In this revised panning procedure, to more efficiently eliminate complexes that bind the solid support non-specifically, we introduced two important modifications. The first modification is the selective elution of target-specific binders from the streptavidin beads (Fig. 1B), achieved by replacing the target protein biotinylation reagent NHS-LC-biotin with NHS-SS-biotin. Since the disulfide linker can be conditionally cleaved with a reducing agent (*e.g.* Tris(2-carboxyethyl)phosphine (TCEP)) with high efficiency, SS-biotin-functionalized target protein and any associated binders can be specifically released from the streptavidin beads, thus separating true binders from non-specific interactors. The second modification is the addition of a new competitive displacement step in which a high concentration of a biotinylated ‘blocker protein’ (*i.e.* BSA-biotin) is used to mask any unoccupied streptavidin molecules on the magnetic beads, displacing non-specific interactors. By combining these two modifications, we have successfully enriched DARPin binders against several clinically relevant targets in as few as three rounds of panning.

### Rapid selection of DARPins specific for IL-10

Recombinant mouse IL-10 was biotinylated with NHS-SS-biotin as described in Materials and Methods. Using a click-displayed naïve DARPin library, we enriched DARPins specific to IL-10 in three successive rounds of panning following the strategy outlined in Table 1. The first round of panning used a target concentration of 100 nM. The second round of panning reduced the target concentration to 50 nM and introduced a negative selection step where the click-displayed library was first incubated with empty streptavidin beads prior to target addition to reduce the abundance of non-specific bead binders. The final round of panning continued using negative selection and further reduced the target concentration to 20 nM. Immediately after target pull-down (see Materials and Methods), the beads were captured by a magnetic stand, resuspended in 250 μl blocking buffer (PTBD0.3, see Materials and Methods) containing 100 nM BSA-biotin blocker protein, and incubated with rotation at room temperature for 15 min to block all unoccupied biotin-binding sites on the streptavidin beads. The beads were then captured and washed the indicated number of times (Table 1) with 250 μl PTBD0.3. After the final wash, the streptavidin beads were incubated in PBS containing 50 mM TCEP to elute the target protein and associated binder-cDNA complexes for PCR amplification. Each round of panning was able to be completed in a single workday. To evaluate the enrichment efficiency, an aliquot of the displayed library from each round of panning was diluted 10-fold in PTBD0.3 with or without biotinylated IL-10 (10 nM final concentration). IL-10 was captured using streptavidin-coated magnetic beads and the relative abundance of DARPin-conjugated cDNA was measured using real-time qPCR. Similar Ct values were observed for all samples incubated in buffer without target, while the Ct values for samples incubated with target progressively decreased after each round of panning (Fig. 2A), indicative of successful enrichment of target-specific binders. A fourth round of panning was also carried out (Fig. 2A), but no further enrichment was seen, so the round 3 library was selected for screening.

**Table 1:** Panning strategies for IL-10 and TGF-β1.

**Target**	**Round**	**PURExpress Scale**	**Target (nM)**	**Strep Bead slurry (μl)**	**Negative Selection**	**# of Washes**
**IL-10**	1	Full	100	25	No	4
	2	Full	50	25	Yes	4
	3	Half	20	10	Yes	6
**TGF-β1**	1	Full	100	25	No	4
	2	Full	100	25	Yes	4
	3	Half	20	10	Yes	6
	4	Half	10	5	Yes	8

**Figure 2:**
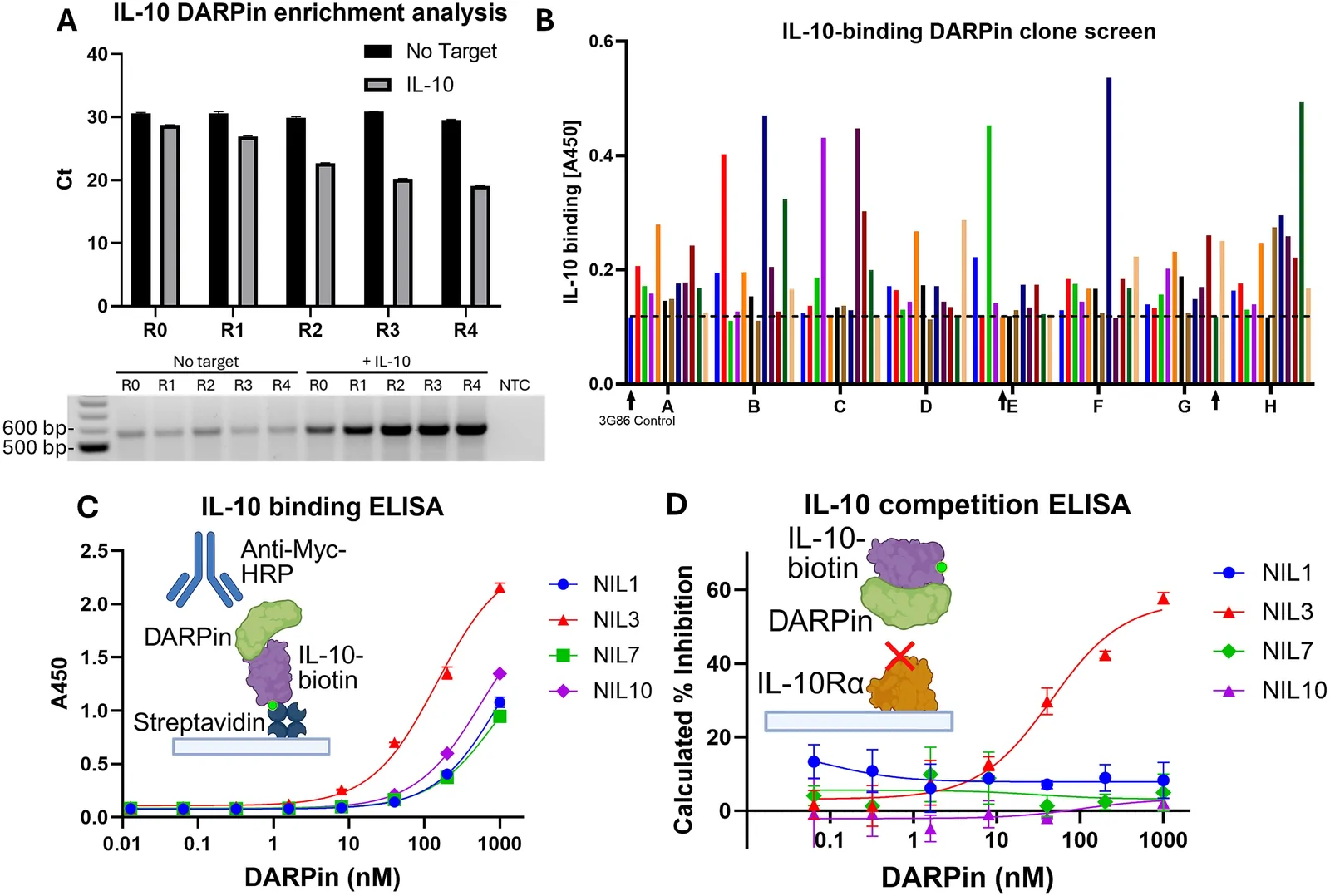
**Rapid selection of DARPins specific to IL-10. (A)** Real-time qPCR of enrichment analysis of click display libraries at each round of panning to IL-10. NTC: No template control. **(B)** Representative graph of lysate ELISA screen from round 3 click display library. The dashed line indicates the average signal of three non-binder DARPin controls (DARPin 3G86, black arrows). See inset of Fig. 2C for diagram of ELISA setup. The X-axis denotes the row of the ELISA plate, and the colored bars represent columns 1–12 within each row. **(C)** ELISA of purified DARPins measuring binding to ELISA plate coated with 20 nM IL-10-biotin. **(D)** Competition ELISA showing calculated percent inhibition of binding of 1 nM IL-10-biotin to ELISA plate coated with 100 μl of 1 μg/ml IL-10Rα. Error bars represent the standard deviation of two technical replicates. ELISA diagrams were generated using BioRender.

The round 3 click display library was subcloned into a pET28 expression vector and transformed into BL21(DE3) *E. coli* cells. A total of 372 clones were selected for screening by lysate ELISA (Fig. 2B). The top 5 clones from each plate (20 total) were selected for sequencing, and of these, 9 unique clones were selected for purification and further screening, of which 4 showed highly specific binding to IL-10 (Supplemental Fig. S1A and B). Clone NIL3 emerged as the strongest binder based on ELISA with an estimated K_d_ of 60.7 nM (R^2^ = 0.9994, K_d_ 95% CI = 50.6 to 73.2) (Fig. 2C). Additionally, NIL3 also inhibited the interaction between IL-10 and its receptor, IL-10Rα, in a dose-dependent manner in a competition ELISA with an estimated IC_50_ of 44.9 nM (R^2^ = 0.965, IC_50_ 95% CI = 22.6 to 91.1) (Fig. 2D).

### Rapid selection of DARPins specific to TGF-**β**1

Recombinant active TGF-β1 (amino acids A279-S390) was biotinylated with NHS-SS-biotin as described in Materials and Methods. DARPins specific to the active domain of TGF-β1 were enriched in four rounds of panning following the strategy outlined in Table 1. Over the course of panning, the target concentration was reduced from 100 nM (Round 1) to 10 nM (Round 4), and negative selection was performed for all rounds except the first round. Immediately after target pull-down, the beads were incubated in BSA-biotin blocking protein (100 nM) for 15 min to mask all unoccupied streptavidin sites. These four rounds of panning were completed in four consecutive workdays. Enrichment analysis showed a steady reduction of the Ct values of samples incubated with the biotinylated target protein and nearly constant Ct values for samples incubated in buffer alone, consistent with the enrichment of binders to TGF-β1 (Fig. 3A).

**Figure 3:**
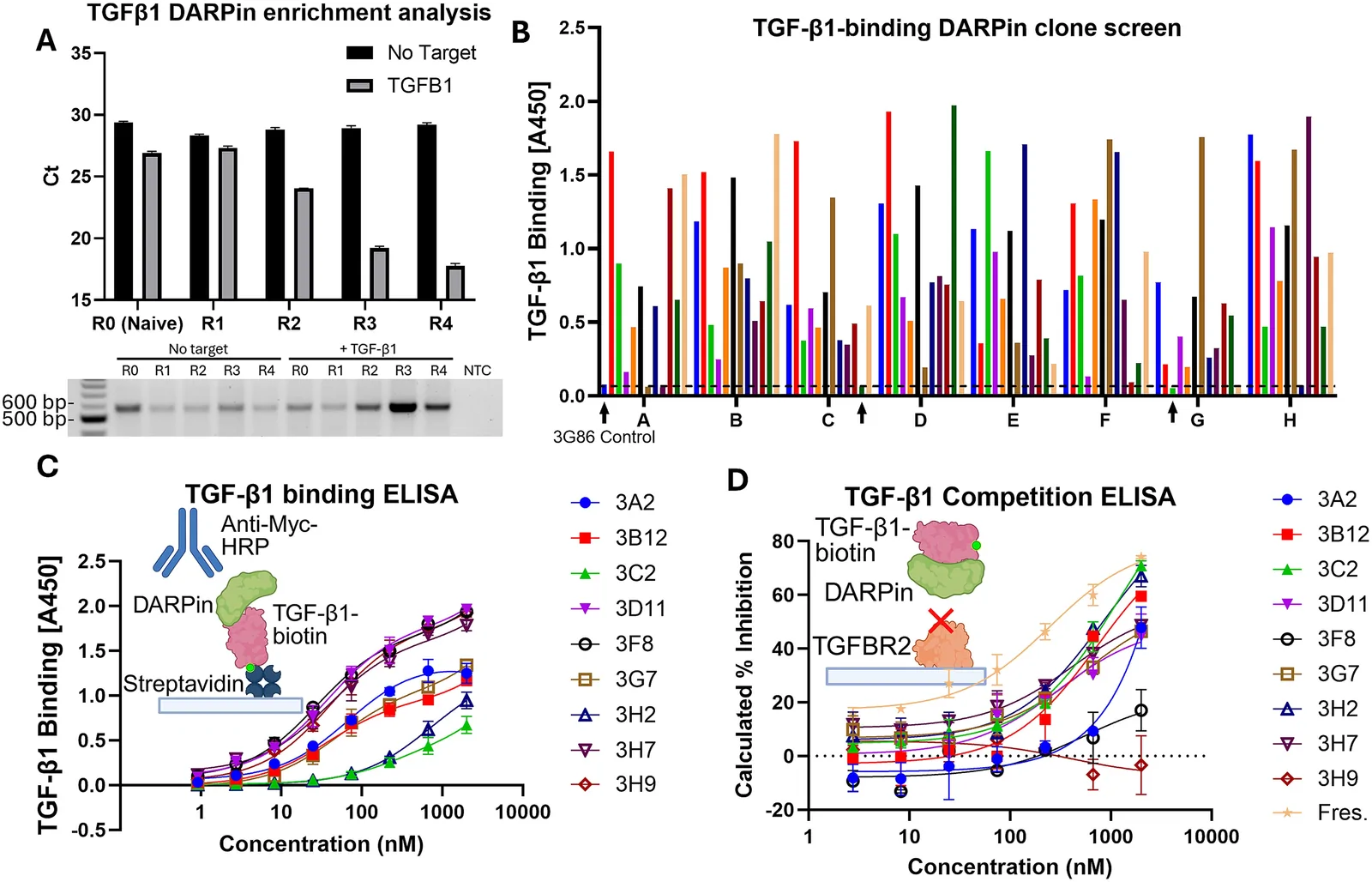
**Rapid selection of DARPins specific to TGF-β1. (A)** Real-time qPCR of enrichment analysis of click display libraries at each round of panning to TGF-β1. NTC: No template control. **(B)** Lysate ELISA screen from round 4 click display library for binders to TGF-β1. The dashed line indicates the average signal of three non-binder DARPin controls (DARPin 3G86, black arrows). See inset of Fig. 3C for diagram of ELISA setup. **(C)** ELISA of purified DARPins to ELISA plate coated with 10 nM TGF-β1-biotin. **(D)** Competition ELISA showing calculated percent inhibition of binding of 5 nM TGF-β1-biotin to ELISA plate coated with 100 μl of 0.5 μg/ml TGFBR2. Error bars represent the standard deviation of two technical replicates. ELISA diagrams were generated using BioRender.

The round 4 click display library was subcloned into a pET28 expression vector and transformed into BL21(DE3) *E. coli* cells. A total of 93 clones were selected for screening by lysate ELISA (Fig. 3B). The top 14 clones were sequenced, and 9 unique clones were purified for further evaluation (Supplemental Fig. S2). The ability of these DARPins to bind specifically to TGF-β1 was confirmed by ELISA (Fig. 3C, Supplemental Fig. S3). To identify DARPins able to inhibit the TGFβ1 signaling pathway, we developed a competition ELISA similar to that described above and measured the ability of DARPins to inhibit the binding of TGF-β1-biotin to ELISA plates coated with TGFBR2, the primary receptor for TGF-β1. Fresolimumab, a pan TGFβ-inhibiting antibody currently undergoing clinical trials (Morris *et al*. 2014, Lacouture *et al*. 2015, Vincenti *et al*. 2017), was used as a positive control. DARPin 3D11 emerged as the most potent TGF-β1 inhibitor (Fig. 3D). Not surprisingly, binding affinity and TGFBR2 inhibition potency did not correlate. For example, 3F8 and 3H9 bind TGF-β1 strongly but did not exhibit any detectable inhibition.

### Affinity maturation of DARPins to PD-L1

Our lab has recently identified a PD-L1-binding DARPin termed A4 using phage display and ELISA screening (Supplemental Fig. S4). A4 only weakly binds to PD-L1, despite being able to dose-dependently inhibit the interaction between PD-L1 and PD-1. We reasoned that by enhancing the affinity of A4 to PD-L1 with affinity maturation we could also improve its effectiveness as an inhibitor. We first codon-optimized the A4 DNA sequence, then used error-prone PCR to generate a randomized library with a predicted average mutation rate of 7 nucleotide changes per gene (Zeng *et al*. 2022). Upon click display, A4 showed minimal binding to biotinylated PD-L1 (Fig. 4A). We then enriched binders against PD-L1 following the conditions outlined in Table 2 with progressively decreasing target protein concentration from 10 nM (Round 1) to 0.4 nM (Round 4). The third round of panning also included an overnight off-rate selection step with a 500-fold molar excess of non-biotinylated PD-L1. Enrichment analysis of the 3^rd^ and 4^th^ rounds of panning against 50 nM of biotinylated PD-L1 showed a strong enrichment of binders compared to the parental A4 DARPin manifested as significantly lower Ct values of samples incubated in the presence of biotin-PD-L1 and the stronger DNA amplification product band on the gel (Fig. 4A).

**Figure 4:**
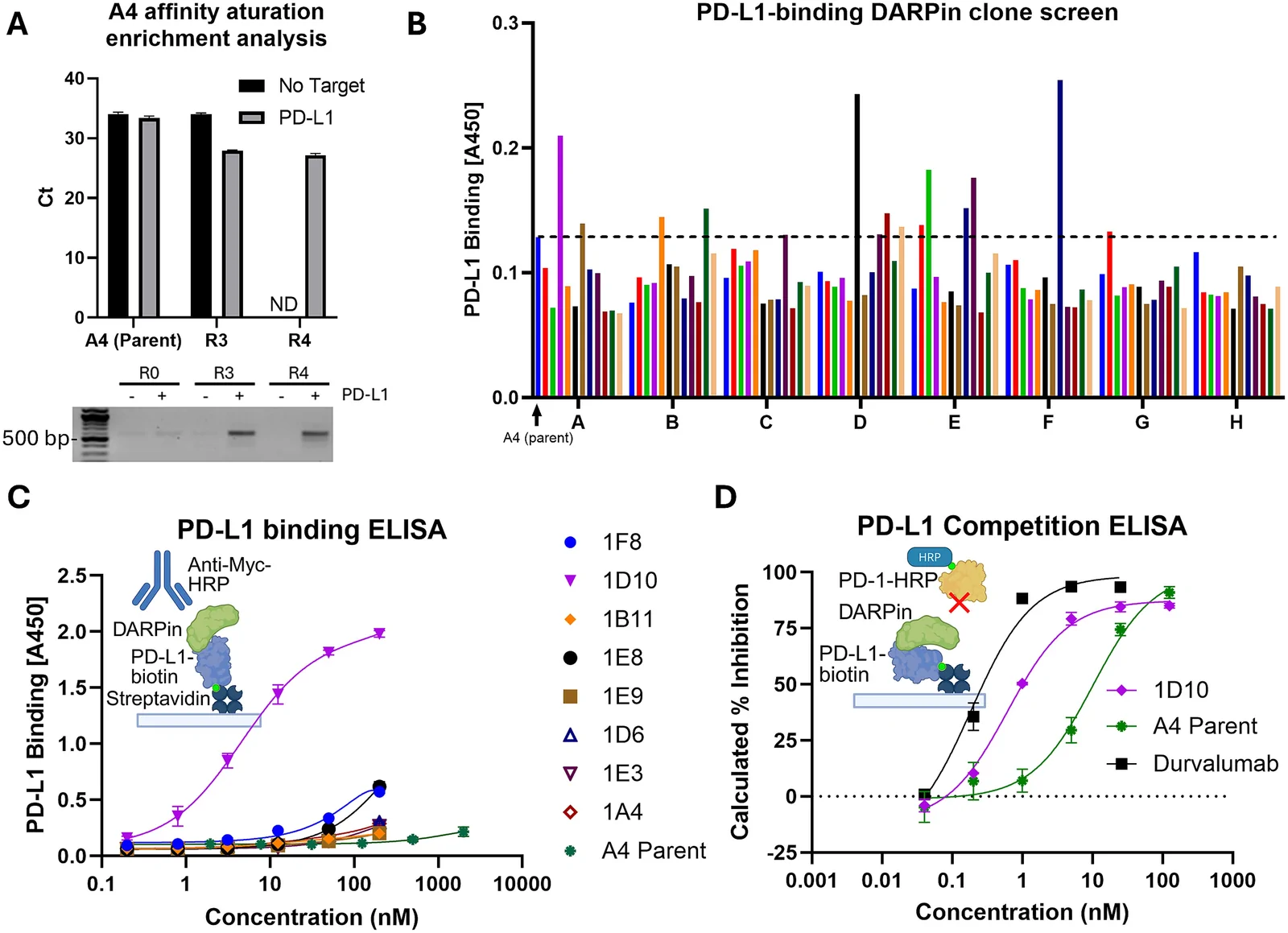
**Affinity maturation of a PD-L1-specific DARPin. (A)** Real-time qPCR of enrichment analysis of click display libraries at rounds 3 and 4 of A4 affinity maturation. ND: Not detected. **(B)** Lysate ELISA screen from round 4 click display library for binders to PD-L1. The dashed line indicates the signal level of the parental A4 DARPin. See inset of Fig. 4C for diagram of ELISA setup. **(C)** ELISA of purified DARPins measuring binding to ELISA plate coated with 20 nM PD-L1-biotin. **(D)** Competition ELISA showing calculated percent inhibition of PD-L1 binding to PD-1. Error bars represent the standard deviation of two technical replicates. ELISA diagrams were generated using BioRender.

**Table 2:** Panning strategy for DARPin affinity maturation.

**Target**	**Round**	**PURExpress Scale**	**Target (nM)**	**Strep Bead slurry (μl)**	**Negative Selection**	**Off-rate selection**	**# of Washes**
**PD-L1**	1	Full	10	5	No	No	4
	2	Full	2	5	No	No	4
	3	Half	2	5	No	1 μM naïve PD-L1 and overnight at RT	4
	4	Half	0.4	5	No	No	8

The round 4 click display library was subcloned into a pET28 expression vector and transformed into BL21(DE3) *E. coli* cells. A total of 95 clones were selected for screening by lysate ELISA (Fig. 4B). Lysates were diluted 120-fold to reduce the A4 signal down to near background levels to enable the identification of better binders. Eight clones were identified as stronger binders and sequenced (Supplemental Fig. S5). We observed an average mutation rate of 4 amino acid changes per molecule among these DARPins. DARPin binding to PD-L1 was measured by ELISA. Although several other DARPins exhibited stronger signal in the lysate ELISA, once purified, 1D10 bound PD-L1 with much higher affinity than all other DARPins tested, with a K_d_ of 4.5 nM (R^2^ = 0.996, K_d_ 95% CI = 3.3 to 6.3), and > 400-fold better than the parent A4 (K_d_ > 2 μM, Fig. 4C). The affinity of 1D10 to biotinylated PD-L1 was measured to be 17.7 nM by BLI (Supplemental Fig. S6). In a competition ELISA, 1D10 showed 17-fold improved PD-1 inhibition potency (IC_50_ = 0.6 nM, R^2^ = 0.996, IC_50_ 95% CI = 0.5 to 0.8) compared to the parent A4 (IC_50_ = 10.2 nM, R^2^ = 0.98, IC_50_ 95% CI = 6.2 to 16.9) (Fig. 4D). This level of inhibition in the competition ELISA was comparable to the IC_50_ we measured for the clinical anti-PD-L1 antibody Durvalumab (Antonia *et al*. 2017) (IC_50_ = 0.2 nM, R^2^ = 0.98, IC_50_ 95% CI = 0.1 to 0.3).

## Discussion

Our lab has previously developed click display – a rapid, facile, and efficient protein display technology – for binder engineering *via* biopanning. Despite early successes, we realized that the overall enrichment rate was significantly slower than conventional phage panning. Additionally, for some target proteins of small size (e.g. <20 kDa, IL-10), we have also encountered difficulty in binder enrichment using the original click display panning method. To improve the panning efficiency, in this paper we have introduced two primary modifications to the biopanning procedure: **i)** selective elution of the target and **ii)** competitive displacement of non-specific binders with a biotinylated blocking protein. We speculate that, by selectively eluting the SS-biotinylated target protein and the associated binders from the streptavidin beads before PCR amplification, we should significantly reduce the likelihood of amplifying non-specific binders to the solid support (i.e. streptavidin beads). Cleavable linkers are advantageous in that they can be used under mild elution conditions, are target specific, and are broadly compatible with many different targets. Previously, disulfide cleavable biotin linkers have been used for phage display (Kobayashi *et al*. 2005), and photocleavable linkers have been used in mRNA and cDNA display (Doi *et al*. 2007) to improve panning efficiency.

Analogous elution strategies have been employed in other cell-free display methods. In ribosome display, mRNA molecules encoding specific target binders are selectively dissociated from the ribosome complexes with EDTA before amplification (Hanes *et al*. 1998, Lipovsek and Pluckthun 2004, Dreier and Plückthun 2012b, 2018). Harsher elution methods have been used with cDNA display, such as elution with low pH (Yamaguchi *et al*. 2009), high pH (Terai *et al*. 2019), or detergents (Terai *et al*. 2019). While these harsher methods are effective in eluting binders from the solid support, they also risk displacing other non-specific binders, potentially reducing the efficiency of enrichment.

Another approach, when available, is to use a target-dependent elution method. For example, when selecting for calcium-dependent calmodulin binders, researchers eluted an mRNA displayed library by chelating calcium with EGTA (Cotten *et al*. 2012). Binders to the anti-FLAG M2 antibody displayed on either mRNA or cDNA were eluted by titrating with a FLAG peptide (Reyes *et al*. 2021). Similarly, streptavidin-binding peptides were displaced with an excess of soluble biotin (Wilson *et al*. 2001). Previously, we have also successfully enriched DARPins able to inhibit fibrinogen binding to ClfA by elution with a high concentration of fibrinogen (Chockalingam *et al*. 2026). Target-specific elution methods have the advantage of being gentle and specific to the target but are also highly dependent on the target itself and are not generally applicable.

It is worth noting that the use of a reducing agent-cleavable biotin linker on the target protein for click display selections has yielded binders with an above-average representation of cysteine residues. In our hands, this phenomenon appeared to be particularly pronounced when our input click-displayed libraries were naïve (*i.e.* fully randomized; Supplemental Figs S1 and S2) rather than starting from a single existing target-binder used as the starting point for affinity maturation (Supplemental Fig. S5). Although the reason for the apparent tendency for selection of cysteine-containing DARPins is unclear; we speculate that the specific target protein or residual DTT from the reverse transcription step could be playing a role. Regardless, in applications that cannot tolerate free-cysteine, more focused libraries constructed from trinucleotide stretches that lack undesirable codons such as those encoding cysteine, proline, and stop codons can be used. Another limitation of using a reducing agent for target/binder elution is it precludes some binder families that require intramolecular disulfide bond for structural stability, such as many antibody formats (e.g. scFv, VHH).

The other significant modification to the click display panning protocol is competitive displacement of non-specific binders with biotinylated BSA. Once the biotinylated target is captured by the streptavidin beads, an excess of biotinylated BSA is added. The biotin/ streptavidin interaction is one of the strongest known ligand-protein interactions and is essentially irreversible under standard conditions (Sano *et al*. 1998, Stayton *et al*. 1999); thus, the biotinylated target will remain securely affixed to the beads. However, the excess biotinylated BSA will bind to any remaining streptavidin on the surface of the beads, coating the binding surface and displacing material that is interacting with the beads non-specifically. Masking these unoccupied streptavidin sites is particularly important during later rounds of enrichment when the target protein is at nanomolar or sub-nanomolar concentrations, making it inevitable that most of the biotin binding sites are unoccupied on the streptavidin beads.

Although we did not carry out side-by-side comparisons to see which of these modifications played the major role, implementing these changes has significantly improved our panning success rate, especially towards small target proteins.

We identified high-affinity, specific binders to three separate targets in only three to four rounds of panning. DARPins that inhibit binding of IL-10 (IC50 = 44.9 nM) and TGF-β1 (IC50 =b  305.6 nM) to their respective receptors were identified from a naïve library. A highly potent PD-L1 inhibitor (IC50 = 0.61 nM) was generated by affinity maturation of a PD-L1-binding DARPin. These proteins were selected for their promise as modulators of immune responses in the tumor microenvironment. The role of IL-10 in tumor development is complex and likely dependent on the type of cancer. In some cases, delivery of exogenous IL-10 is antitumorigenic, activating CD8^+^ T cells and enhancing IFN-γ production (Oft 2014, Qiao *et al*. 2019, Salkeni and Naing 2023 Jun 14). In contrast, IL-10 blockade with an IL-10 antibody induced apoptosis in CRLM samples (Sullivan *et al*. 2023) and reduced tumor size in a B16F10 melanoma model (Gordy *et al*. 2018), suggesting a role for IL-10 inhibitors in certain cancer types. TGF-β also appears to have a dual role in cancer development, acting as a tumor suppressor early but promoting immune evasion and metastasis in advanced cancers (Batlle and Massagué 2019 Apr 16). Similarly, the PD-1/PD-L1 signaling pathway is an important immune checkpoint that cancer cells can exploit by increasing surface expression of PD-L1 (Pardoll 2012, Sharpe and Pauken 2018). Preclinical studies suggest that inhibition of TGF-β alone may have limited effectiveness, but the combination of TGF-β inhibitors and PD-L1 checkpoint inhibitors acts synergistically to improve the effectiveness of both treatments (Holmgaard *et al.* 2018 Jun 4, Mariathasan *et al*. 2018, Nakanishi *et al*. 2018).

In summary, we demonstrate an optimized click display panning protocol that enables rapid and robust enrichment of high quality target binders through a simple, entirely *in vitro* workflow. By combining selective target elution with competitive suppression of bead-associated background, the platform overcomes a major limitation of earlier click display panning and supports efficient discovery of functional binders from naïve libraries. Using this approach, we identified receptor-blocking DARPins against IL-10 and TGF-β1 and generated a markedly improved PD-L1 inhibitor through affinity maturation, highlighting the versatility of the method across distinct targets and engineering objectives. Given its speed, scalability, and lack of transformation requirement, our optimized click display panning protocol should be broadly useful for accelerating binder discovery, affinity maturation, and future directed-evolution campaigns in therapeutic and diagnostic development.

## Materials and methods

### Target protein biotinylation

Target proteins were biotinylated using EZ-Link NHS-SS-Biotin (Thermo Scientific 21 441) in PBS with a 1–3-h incubation at room temperature followed by overnight incubation at 4°C. IL-10 (Sino Biological 50 245-MNAE) and TGF-β1 (Sino Biological 10 804-HNAC) were biotinylated at a 1:4 target:biotin molar ratio, and PD-L1 (GenScript Z03425) and PD-1 (GenScript Z03424) were biotinylated at a 1:10 ratio. The BSA blocker protein was biotinylated using EZ-Link Sulfo-NHS-LC-Biotin (Thermo Scientific 21 335) at a 1:20 ratio. Excess unreacted biotin was removed by passing the reaction mixture through two consecutive 7 K Zeba columns (Thermo Scientific 89 882).

### Click display procedure

DARPin libraries were displayed essentially as we have described previously (Zeng *et al*. 2023), with some minor modifications. A full-scale display reaction consisted of 10 μl Solution A and 7.5 μl Solution B from the PURExpress *In Vitro* Protein Synthesis kit (NEB E6800), 2 μl of 25 μM modified linker (ML, described in Zeng *et al* (Zeng *et al*. 2023)), 0.5 μl murine RNase inhibitor (NEB M0314), and 5 μl library DNA (200 ng/μl, naïve library synthesis is described in (Zeng *et al*. 2023)) (Supplemental Fig. S7). For half-scale reactions, all volumes were halved. This reaction mixture was incubated at 37°C for 20 min followed by UV irradiation for 30 s under a UV lamp (SUNUV Gel Nail Light, Model SUNONEWT) to crosslink the ML to the mRNA. Transcription was terminated by adding 2 μl XbaI (NEB R0145) and incubating at 37°C for 10 min. The reaction was then placed on an orbital shaker (VWR, Symphony DS41) shaking at 1200 rpm at 37°C for 30 min, followed by incubation at room temperature (RT) for 20 min without shaking. cDNA synthesis was performed by setting up a reverse transcription reaction containing the click display library, 1× SuperScript IV RT buffer, 5 mM DTT, 0.5 mM dNTPs, and 400 U SuperScript IV RT (Invitrogen 18090010) in a final volume of 150 μl, then incubating at 50°C for 30 min. Double-stranded input DNA was cleaved by adding 0.5 μl KpnI and incubating at 37°C for 30 min. Reactions were desalted into PBS by passing through a 40 K Zeba column (Thermo Scientific A57760). An aliquot of the displayed library was stored at −80°C for future analysis.

### Library panning

Specific details for each round of panning for each target can be found in Tables 1 and 2, but the general panning procedure is as follows (see also Fig. 1). All tubes used during panning were pre-blocked for at least 30 min with blocking buffer PTBD0.3 (1× PBS (Fisher Scientific BP39920), 0.1% Tween-20, 2% BSA, and 0.3 mg/ml salmon sperm DNA). Magnetic streptavidin beads (Dynabeads MyOne Streptavidin T1, Invitrogen 65 601) were prepared by washing three times with PTBD0.3 then blocking in PTBD0.3 for at least 30 min. After buffer exchange to remove DTT, the click-displayed library was diluted 5-fold in PTBD0.3 containing the biotinylated target protein and incubated for 1 h at RT with rotation. When negative selection was used, the click display library was pre-incubated with 25 μl blocked streptavidin bead slurry for 30 min, then the beads were captured and the supernatant containing click display library was transferred to a fresh tube (blocked with PTBD0.3) prior to the addition of target. After target incubation, fresh streptavidin beads (blocked with PTBD0.3) were added and the mixture was incubated at RT for 30 min with rotation to promote pull-down. The beads were captured on a magnetic stand and resuspended in 250 μl PTBD0.3 supplemented with 100 nM BSA-biotin blocker protein to occupy all empty streptavidin sites, then incubated at RT with rotation for 15 min. The beads were then washed the indicated number of times with 250 μl PTBD0.3. On the final wash step, the beads were transferred to a fresh tube. The supernatant was completely removed, and the beads were then incubated in 20 μl PBS containing 50 mM TCEP to elute the target protein and the associated library. The library was then PCR-amplified using Q5 high fidelity DNA polymerase (NEB M0491) with primers 1938 (5′- ATACGAAATTAATACGACTCACTATAGGGAGACCACAA CGGTTTCCCTCTAGAAATAATTTTGTTTAAC-3′) and 2643 (5′- AAACCCCTCCGTTTA GAGAGGGGTTATGCTAGTTATTCCACGCCGCCC-CCCGTCCT-3′). The correct DNA band was gel purified (Monarch Spin DNA gel extraction kit, NEB T1120) and used in the next round of panning.

### Enrichment analysis

An aliquot of displayed library (*i.e.* 15 μl) from each round of panning was diluted 10-fold in blocking buffer (25–30 μl per sample) with or without biotinylated target (10 nM IL-10, 10 nM TGF-β1, or 50 nM PD-L1). After incubating for 1 h at RT with rotation, the target was captured using streptavidin-coated magnetic beads (5 μl slurry). After capture, the beads were washed eight times with PTBD0.3 and transferred to fresh tubes for a final wash step. Binders were eluted from the beads using 20 μl PBS with 50 mM TCEP as described above. The relative abundance of eluted cDNA was measured by real-time qPCR using Forget-MeNot EvaGreen qPCR Master Mix (Biotium, 31045) in a qPCR instrument (Bio-Rad, CFX Duet Real-Time PCR Detection Systems). The primers 2900 (5′- GCCACCATCACCACCATCAT-3′) and 2908 (5′- TTGAGGAGAGTAGGACCTCCG-3′) were used for TGF-β1 and IL-10, and primers 1892 (5′- TTGAGGAGAGTAGGACCT-3′) and 2488 (5′- GTTTAACTTTAAG AAGGAGGATATATCCAT-3′) were used for PD-L1. qPCR reactions were performed in technical duplicates.

### ELISA experiments

Lysate ELISA screens: Enriched libraries from the final round of panning for each target were subcloned into a pET28 expression vector and transformed into *Escherichia coli* BL21 (DE3) cells. Single isolated clones were used to inoculate individual wells of a deep well 96 well plate containing 1 ml LB medium, and expression was induced with 0.5 mM IPTG when the average OD_600_ reached ~0.5. Four hours post-induction, cells were pelleted and the growth medium was removed. Cells were lysed with 4 freeze/thaw cycles in 100 μl/well lysis buffer (containing 1× PBS, 200 μg/ml lysozyme, 1 mM CaCl_2_, 0.5 mM EDTA, 2.5 mM MgCl_2_, and 25 U/ml benzonase). MaxiSorp ELISA plates (Thermo Scientific 442404) were coated with 100 μl of 4 μg/ml streptavidin in PBS for 1 h at RT then blocked with PBS-TB (1× PBS, 2% BSA, 0.1% Tween-20) at RT for 1 h. Plates were then coated with 100 μl/well biotinylated target (30 nM IL-10, 10 nM TGF-β1, or 50 nM PD-L1). Coated ELISA plates were incubated with 100 μl/well diluted lysate (10-fold dilution for IL-10 and TGF-β1 clones and 120-fold dilution for PD-L1). All DARPins contained Myc- and 8× His-tags, thus binding DARPins were detected with 1:20000 chicken anti-Myc-HRP (Immunology Consultants Laboratory cmyc-45P-Z) and color development with TMB (3,3′, 5,5′′-tetramethylbenzidine) colorimetric substrate. Absorbance was measured at 450 nm.

Purified DARPin ELISA: ELISAs with IMAC purified DARPins were performed essentially as described above, except multiple concentrations of DARPin were used, as indicated.

IL-10 and TGF-β1 Competition ELISAs: MaxiSorp ELISA plates were coated with 100 μl of either 1 μg/ml IL-10Rα (Sino Biological 51 298-M02H) or 0.5 μg/ml TGFBR2 (Sino Biological 10 358-H02H) for 1 h at RT then blocked with PBS-TB as described above. DARPins were serially diluted in a separate blocked 96-well plate (non-ELISA) then mixed with their respective target (1 nM IL-10-biotin or 2.5 nM TGF-β1-biotin). After a 30-min incubation, the DARPin/target mixture was transferred to the blocked ELISA plate and incubated for 1 h. Biotinylated target was detected with a 1:100000 dilution of streptavidin-HRP and color was developed with TMB. Percent inhibition was calculated using the formula 100×[1-(A450_DARPin_/A450_Max_)], where A450_DARPin_ is the absorbance at 450 nm at a given DARPin concentration and A450_Max_ is the average signal across four technical replicates in the absence of DARPin.

PD-L1 Competition ELISA: MaxiSorp ELISA plates were coated with 100 μl of 4 μg/ml streptavidin for 1 h at RT then blocked with PBS-TB containing 0.2% BSA at 4°C overnight. Wells were then coated with 100 μl of 20 nM PD-L1-biotin. DARPins were serially diluted in a separate blocked 96-well plate (non-ELISA) then 50 μl of each dilution was transferred to corresponding wells of the ELISA plate coated with PD-L1. PD-1-HRP was prepared by combining PD-1-biotin with Strep-HRP at a 5:4 molar ratio and incubating for 30 min at RT. PD-1-HRP was diluted to 20 nM in PBS-TB containing a 10-fold molar excess (200 nM) of free biotin to mask any remaining unconjugated Streptavidin. 50 μl of PD-1-HRP was added to the DARPins on each well of the ELISA plate then the plate was incubated for 1 h at RT. After thorough washing, color was developed with TMB, and absorbance was measured at 450 nm. Percent inhibition was calculated using the formula 100×[1-(A450_DARPin_/A450_Max_)], where A450_DARPin_ is the absorbance at a given DARPin concentration and A450_Max_ is the average signal across four technical replicates in the absence of DARPin.

### DARPin expression and purification

All DARPin constructs were cloned downstream of N-terminal 6xHis and c-myc tags in a pET28 expression vector and expressed in *E. coli* BL21 (DE3) cells in either TB (Terrific Broth) or LB medium supplemented with kanamycin (50 μg/ml). In a typical DARPin expression experiment, the overnight culture was diluted at least 50-fold in growth media and induced with 0.5 mM Isopropyl-β-D-1-thiogalactopyranoside (IPTG) when the OD_600_ reached ~0.6 (for LB) or ~1.2 (for TB). The culture was either transferred to an 18°C incubator and grown overnight at 250 rpm or continued to grow at 37°C/250 rpm for 4 h before harvesting. The cell pellets were resuspended in Base Buffer (50 mM NaPO_4_, pH 8.0, 300 mM NaCl) and lysed using B-PERII Bacterial Protein Extraction Reagent (ThermoFisher 78260). All proteins were purified using PureCube Ni-NTA agarose resin (Cube Biotech 74105) following a standard protocol (Woolley *et al*. 2026). Protein concentration was measured by BCA assay (Thermo Scientific 23225) with BSA standard, and purity was determined using 12% or 4%–20% SDS-PAGE gels.

### Error-prone PCR of DARPin A4

The error-prone PCR reaction contained 100 mM Tris–HCl, 500 mM KCl, 70 mM MgCl_2_, 0.1% (w/v) BSA, 500 μM MnCl_2_, 200 μM dGTP/dATP, 1 mM dCTP/dTTP, 200 nM primers 1938 and 2643, 20 ng/μl A4 plasmid template, and 50 U/ml Taq DNA polymerase. Following 25 cycles of PCR, the amplicon was gel purified before being used for click display.

## Supplementary Material

Supplement_ZC_082826_gzag028

## Abbreviations

DARPin, designed ankyrin repeat protein; TCEP, Tris(2-carboxy-ethyl)phosphine

## Data analysis

ELISA binding data were fit to the One site – Total binding model in GraphPad Prism to determine K_d_. Competition ELISA data were fit to the [Inhibitor] *vs.* response (three parameters) model in GraphPad Prism to determine IC50.

## References

[ref1] Antonia SJ, Villegas A, Daniel D et al. Durvalumab after chemoradiotherapy in stage III non–small-cell lung cancer. N Engl J Med 2017;377:1919–29. 10.1056/nejmoa1709937. 28885881

[ref2] Batlle E, Massagué J. Transforming grown factor-β Signaling in immunity and cancer. Immunity 2019;50:924–40. 10.1016/j.immuni.2019.03.024. PMC7507121 30995507

[ref3] Boder ET, Wittrup KD. In: Jeremy Thorner SDE and John NA (eds.), *Method Enzymol*: Academic Press, 2000, pp. 430–44. 10.1016/S0076-6879(00)28410-3. 11075358

[ref4] Bowers PM, Horlick RA, Neben TY et al. Coupling mammalian cell surface display with somatic hypermutation for the discovery and maturation of human antibodies. Proc Natl Acad Sci U S A 2011;108:20455–60. 10.1073/pnas.1114010108. PMC3251138 22158898

[ref5] Chao G, Lau WL, Hackel BJ et al. Isolating and engineering human antibodies using yeast surface display. Nat Protoc 2006;1:755–68. 10.1038/nprot.2006.94. 17406305

[ref6] Chockalingam K, Banerjee B, Zeng Y et al. Dual-mode ClfA-targeting DARPin biologics protect against diverse methicillin-resistant Staphylococcus aureus strains. bioRxiv, 2026.2001.2009.698026. First published on 20260112 2026 10.64898/2026.01.09.698026.

[ref7] Chonira V, Kwon YD, Gorman J et al. A potent and broad neutralization of SARS-CoV-2 variants of concern by DARPins. Nat Chem Biol 2023;19:284–91. 10.1038/s41589-022-01193-2. PMC10294592 36411391

[ref8] Clackson T, Hoogenboom HR, Griffiths AD et al. Making antibody fragments using phage display libraries. Nature 1991;352:624–8. First published on. 10.1038/352624a0. 1907718

[ref9] Cotten SW, Zou J, Wang R et al. mRNA display based selections using synthetic peptide and natural protein libraries. Methods Mol Biol 2012;805:287–97. 10.1007/978-1-61779-379-0_16. PMC4259390 22094812

[ref10] Daugherty PS . Protein engineering with bacterial display. Curr Opin Struct Biol 2007;17:474–80. First published on 20070828. 10.1016/j.sbi.2007.07.004. 17728126

[ref11] Doi N, Takashima H, Wada A et al. Photocleavable linkage between genotype and phenotype for rapid and efficient recovery of nucleic acids encoding affinity-selected proteins. J Biotechnol 2007;131:231-239. 10.1016/j.jbiotec.2007.07.947. 17767970

[ref12] Doi N, Yanagawa H. Genotype-phenotype linkage for directed evolution and screening of combinatorial protein libraries. Comb Chem High Throughput Screen 2001;4:497-509. 10.2174/1386207013330878. 11562254

[ref13] Dreier B, Plückthun A. Rapid selection of high-affinity antibody scFv fragments using ribosome display. Methods Mol Biol 2018;1827:235–68. 10.1007/978-1-4939-8648-4_13. 30196501

[ref14] Dreier B, Plückthun A. Rapid selection of high-affinity binders using ribosome display. Methods Mol Biol 2012a;805:261–86. 10.1007/978-1-61779-379-0_15. 22094811

[ref15] Dreier B, Plückthun A. Ribosome display: A technology for selecting and evolving proteins from large libraries. Methods Mol Biol 2011;687:283–306. 10.1007/978-1-60761-944-4_21. 20967617

[ref16] Dreier B, Plückthun A. In: Douthwaite JA and Jackson RH (eds.), *Ribosome Display and Related Technologies: Methods and Protocols*. New York, NY: Springer, 2012b, pp. 261–86.

[ref17] Gordy JT, Luo K, Francica B et al. Anti-IL-10–mediated enhancement of antitumor efficacy of a dendritic cell–targeting MIP3α-gp100 vaccine in the B16F10 mouse melanoma model is dependent on type I interferons. J Immunother 2018;41:181–9. 10.1097/cji.0000000000000212. PMC5891382 29334492

[ref18] Hanes J, Jermutus L, Weber-Bornhauser S et al. Ribosome display efficiently selects and evolves high-affinity antibodies *in vitro* from immune libraries. Proc Natl Acad Sci 1998;95:14130–5. 10.1073/pnas.95.24.14130. PMC24338 9826665

[ref19] Ho M, Nagata S, Pastan I. Isolation of anti-CD22 Fv with high affinity by Fv display on human cells. Proc Natl Acad Sci U S A 2006;103:9637–42. 10.1073/pnas.0603653103. PMC1480459 16763048

[ref20] Holmgaard RB, Schaer DA, Li Y et al. Targeting the TGFβ pathway with galunisertib, a TGFβRI small molecule inhibitor, promotes anti-tumor immunity leading to durable, complete responses, as monotherapy and in combination with checkpoint blockade. J Immunother Cancer 2018;6:47. 10.1186/s40425-018-0356-4. PMC5987416 29866156

[ref21] Kobayashi N, Karibe T, Goto J. Dissociation-independent selection of high-affinity anti-hapten phage antibodies using cleavable biotin-conjugated haptens. Anal Biochem 2005;347:287–96. First published on 20051006. 10.1016/j.ab.2005.09.016. 16288966

[ref22] Lacouture ME, Morris JC, Lawrence DP et al. Cutaneous keratoacanthomas/squamous cell carcinomas associated with neutralization of transforming growth factor β by the monoclonal antibody fresolimumab (GC1008). Cancer Immunol Immunother 2015;64:437–46. 10.1007/s00262-015-1653-0. PMC6730642 25579378

[ref23] Leemhuis H, Stein V, Griffiths AD et al. New genotype–phenotype linkages for directed evolution of functional proteins. Curr Opin Struc Biol 2005;15:472–8. 10.1016/j.sbi.2005.07.006. 16043338

[ref24] Lipovsek D, Pluckthun A. In-vitro protein evolution by ribosome display and mRNA display. J Immunol Methods 2004;290:51–67. 10.1016/j.jim.2004.04.008. 15261571

[ref25] Mariathasan S, Turley SJ, Nickles D et al. TGFβ attenuates tumour response to PD-L1 blockade by contributing to exclusion of T cells. Nature 2018;554:544–8. 10.1038/nature25501. PMC6028240 29443960

[ref26] Mathonet P, Ioannou A, Betley J et al. CIS display, a DNA-based selection technology for therapeutic peptides. Chim Oggi 2011;29:10–2.

[ref27] Morris JC, Tan AR, Olencki TE et al. Phase I study of GC1008 (fresolimumab): A human anti-transforming growth factor-beta (TGFβ) monoclonal antibody in patients with advanced malignant melanoma or renal cell carcinoma. PLoS One 2014;9:e90353. 10.1371/journal.pone.0090353. PMC3949712 24618589

[ref28] Naimuddin M, Kubo T. A high performance platform based on cDNA display for efficient synthesis of protein fusions and accelerated directed evolution. ACS Comb Sci 2016;18:117–29. 10.1021/acscombsci.5b00139. 26812183

[ref29] Nakanishi Y, Duran A, L’Hermitte A et al. Simultaneous loss of both atypical protein kinase C genes in the intestinal epithelium drives serrated intestinal cancer by impairing immunosurveillance. Immunity 2018;49:1132–1147.e7. 10.1016/j.immuni.2018.09.013. PMC6301096 30552022

[ref30] Newton MS, Cabezas-Perusse Y, Tong CL et al. In vitro selection of peptides and proteins-advantages of mRNA display. ACS Synth Biol 2020;9:181–90. 10.1021/acssynbio.9b00419. PMC8203280 31891492

[ref31] Odegrip R, Coomber D, Eldridge B et al. CIS display: In vitro selection of peptides from libraries of protein-DNA complexes. Proc Natl Acad Sci U S A 2004;101:2806–10. 10.1073/pnas.0400219101. PMC365701 14981246

[ref32] Oft M . IL-10: Master switch from tumor-promoting inflammation to antitumor immunity. Cancer Immunol Res 2014;2:194–9. 10.1158/2326-6066.cir-13-0214. 24778315

[ref33] Pardoll DM . The blockade of immune checkpoints in cancer immunotherapy. Nat Rev Cancer 2012;12:252–64. 10.1038/nrc3239. PMC4856023 22437870

[ref34] Peng Z, Simeon R, Mitchell SB et al. Designed ankyrin repeat protein (DARPin) neutralizers of TcdB from Clostridium difficile Ribotype 027. mSphere 2019;4 10.1128/mSphere.00596-19. PMC6796971 31578248

[ref35] Plückthun A . Ribosome display: A perspective. Methods Mol Biol 2012;805:3–28. 10.1007/978-1-61779-379-0_1. 22094797

[ref36] Qiao J, Liu Z, Dong C et al. Targeting Tumors with IL-10 prevents dendritic cell-mediated CD8+ T cell apoptosis. Cancer Cell 2019;35:901–915.e4. 10.1016/j.ccell.2019.05.005. 31185213

[ref37] Reyes SG, Kuruma Y, Fujimi M et al. PURE mRNA display and cDNA display provide rapid detection of core epitope motif via high-throughput sequencing. Biotechnol Bioeng 2021;118:1736–49. 10.1002/bit.27696. 33501662

[ref38] Roberts RW, Szostak JW. RNA-peptide fusions for the in vitro selection of peptides and proteins. Proc Natl Acad Sci U S A 1997;94:12297–302. First published on. 10.1073/pnas.94.23.12297. PMC24913 9356443

[ref39] Rutherford N, Mourez M. Surface display of proteins by gram-negative bacterial autotransporters. Microb Cell Factories 2006;5:22. 10.1186/1475-2859-5-22. PMC1533851 16787545

[ref40] Salkeni MA, Naing A. Interleukin-10 in cancer immunotherapy: From bench to bedside. Trends Cancer 2023;9:716–25. 10.1016/j.trecan.2023.05.003. PMC10524969 37321942

[ref41] Sano T, Vajda S, Cantor CR. Genetic engineering of streptavidin, a versatile affinity tag. J Chromatogr B Biomed Sci Appl 1998;715:85–91. 10.1016/S0378-4347(98)00316-8. 9792500

[ref42] Schaffitzel C, Hanes J, Jermutus L et al. Ribosome display: An in vitro method for selection and evolution of antibodies from libraries. J Immunol Methods 1999;231:119–35. 10.1016/S0022-1759(99)00149-0. 10648932

[ref43] Sharpe AH, Pauken KE. The diverse functions of the PD1 inhibitory pathway. Nat Rev Immunol 2018;18:153–67. 10.1038/nri.2017.108. 28990585

[ref44] Shusta EV, Holler PD, Kieke MC et al. Directed evolution of a stable scaffold for T-cell receptor engineering. Nat Biotechnol 2000;18:754–9. 10.1038/77325. 10888844

[ref45] Simeon R, Jiang M, Chamoun-Emanuelli AM et al. Selection and characterization of ultrahigh potency designed ankyrin repeat protein inhibitors of C. Difficile toxin B. PLoS Biol 2019;17:e3000311. 10.1371/journal.pbio.3000311. PMC6590788 31233493

[ref46] Stayton PS, Freitag S, Klumb LA et al. Streptavidin–biotin binding energetics. Biomol Eng 1999;16:39–44. 10.1016/S1050-3862(99)00042-X. 10796983

[ref47] Sullivan KM, Jiang X, Guha P et al. Blockade of interleukin 10 potentiates antitumour immune function in human colorectal cancer liver metastases. Gut 2023;72:325–37. 10.1136/gutjnl-2021-325808. PMC9872249 35705369

[ref48] Terai T, Anzai H, Nemoto N. Selection of peptides that associate with dye-conjugated solid surfaces in a pH-dependent manner using cDNA display. ACS Omega 2019;4:7378–84. 10.1021/acsomega.9b00631. PMC6649003 31459836

[ref49] Ueno S, Kimura S, Ichiki T et al. Improvement of a puromycin-linker to extend the selection target varieties in cDNA display method. J Biotechnol 2012;162:299–302. 10.1016/j.jbiotec.2012.09.003. 23000082

[ref50] Valencia CA, Zou J, Liu R. In vitro selection of proteins with desired characteristics using mRNA-display. Methods 2013;60:55–69. 10.1016/j.ymeth.2012.11.004. PMC3877681 23201412

[ref51] Vincenti F, Fervenza FC, Campbell KN et al. A phase 2, double-blind, placebo-controlled, randomized study of Fresolimumab in patients with steroid-resistant primary focal segmental glomerulosclerosis. Kidney Int Rep 2017;2:800–10. 10.1016/j.ekir.2017.03.011. PMC5733825 29270487

[ref52] Wilson DS, Keefe AD, Szostak JW. The use of mRNA display to select high-affinity protein-binding peptides. Proc Natl Acad Sci 2001;98:3750–5. 10.1073/pnas.061028198. PMC31124 11274392

[ref53] Woolley M, Bryant JD, Vrana S et al. Repurpose an FDA-approved antibody using DARPin-scaffolded bridge protein. Chemistry–Methods 2026;6:e70098. 10.1002/cmtd.70098.

[ref54] Yamaguchi J, Naimuddin M, Biyani M et al. cDNA display: A novel screening method for functional disulfide-rich peptides by solid-phase synthesis and stabilization of mRNA–protein fusions. Nucleic Acids Res 2009;37:e108–8. 10.1093/nar/gkp514. PMC2760808 19528071

[ref55] Zahnd C, Amstutz P, Plückthun A. Ribosome display: Selecting and evolving proteins in vitro that specifically bind to a target. Nat Meth 2007;4:269–79. 10.1038/nmeth1003. 17327848

[ref56] Zeng Y, Jiang M, Robinson S et al. A multi-specific DARPin potently neutralizes Shiga toxin 2 via simultaneous modulation of both toxin subunits. Bioengineering (Basel) 2022;9 10.3390/bioengineering9100511. PMC9598796 36290479

[ref57] Zeng Y, Woolley M, Chockalingam K et al. Click display: A rapid and efficient in vitro protein display method for directed evolution. Nucleic Acids Res 2023;51:e89–9. 10.1093/nar/gkad643. PMC10484664 37548398

[ref58] Zhou C, Jacobsen FW, Cai L et al. Development of a novel mammalian cell surface antibody display platform. MAbs 2010;2:508–18. 10.4161/mabs.2.5.12970. PMC2958572 20716968

