# Supplementary material for: Rapid selection of high-affinity DARPins using click display
Source: Protein Eng Des Sel. 2026 Oct 3;39:gzag028. doi: 10.1093/protein/gzag028 (PMC13654297; doi:10.1093/protein/gzag028)
Supplement: Supplementary file 1 [file supplement_zc_082826_gzag028.docx]

**Supplementary Information.**

**Supplemental Methods**

Engineering of DARPin A4 against PD-L1

Using an in-house DARPin library displayed on M13 phage, we carried out three rounds of phage panning against biotinylated PD-L1 (SinoBio, 10084-H08H). For the phage ELISA, Nunc MaxiSorp ELISA plates were coated with 4 µg/mL streptavidin or neutravidin in PBS for 1 hour at room temperature with rocking, then blocked overnight with blocking buffer (PBS, 0.1% Tween-20, 2% BSA), and then coated with 20 nM biotinylated PD-L1. After washing, 5 x 10^11^ phage particles (diluted in blocking buffer) harvested from each round of panning were added to the wells and incubated for 1 hour at room temperature with rocking. After thorough washing, phage particles remaining in each well were detected using an anti-M13 antibody conjugated to horseradish peroxidase (HRP) (Thermo Scientific) and BioFX TMB One Component HRP Substrate (VWR)(Supplemental Figure 4).

The final round of enriched DARPin pool was cloned into a pET28 expression vector and transformed into BL21(DE3) cells. A total of 93 colonies were grown in 96-well V-bottom plate in LB supplemented with kanamycin (final 50 µg/mL, 100 µL/well) overnight at 37 ºC and 600 rpm. The next day, 50 µL of the overnight culture was transferred to a fresh V-bottom 96-deep well plate containing 950 µL of LB (supplemented with 50 µg/mL kanamycin) and grown at 37 ºC at 600 rpm until average OD600 reached ~0.6, at which point protein expression was induced with 0.5 mM IPTG. The plates were grown at 37 ºC at 600 rpm for an additional 4 hours, after which the cells were harvested by centrifugation. Cell lysate was prepared by resuspending each pellet in 200 µL lysis buffer [PBS, 200 µg/mL lysozyme (Thermo 89833), 1 mM CaCl_2_, 0.5 mM EDTA, 25 µg/mL Benzonase (Millipore, 70746-4)] and incubated at 37 ºC for 30 minutes followed by three freeze-thaw cycles (30 minutes each at -80 ºC and 37 ºC). Crude lysate was clarified by centrifugation. Clones underwent duplicate ELISA screen for those i) able to bind biotinylated PD-L1 and ii) show reduced binding in the presence of B7-H1, an anti-PD-L1 blocking antibody. Due to the relatively weak affinity of PD-1, B7-H1 (Sinobio 10084-R639) was used as a surrogate to identify binders able to bind the same epitope as PD-1. Briefly, duplicate ELISA plates were coated with 80 µL PD-L1 (3 µg/mL in PBS) at 4 ºC. The next day, the ELISA plates were blocked with 300 µL/well blocking buffer (PBS, 0.1% Tween 20, 2% BSA) at room temperature for 1 hour. For the binding ELISA, 10 µL of soluble lysate and 80 µL blocking buffer were added to each well. For competition ELISA, 20 µL lysate and 50 µL B7-H1 (75 nM in PBS) were added. After thorough washing in PBST (PBS, 0.1% Tween-20), immobilized DARPin was quantified using chicken anti-Myc antibody-HRP (Immunology Consultants Laboratory cmyc-45P-Z) and TMB (3,3′, 5,5′′-tetramethylbenzidine) colorimetric substrate. Absorbance was measured at 450 nm. Eight clones showing an above-background PD-L1 binding signal and >20% reduced binding signal in the presence of B7-H1 were selected. Among these, A4 emerged as the most promising candidate.

**Supplemental Figure S1A** – IL-10 -binding DARPin sequences

| Motif | NCAP / AR1 / AR2 / AR3 / CCAP / |
| --- | --- |
| Clone | GKKLLEAARAGQDDEVRILMANGADVNA/XXXXGXTPLHLAAXXGHLEIVEVLLKXGADVNA/XXXXGXTPLHLAAXXGHLEIVEVLLKXGADVNA/XXXXGXTPLHLAAXXGHLEIVEVLLKXGADVNA/QXXXGXTAFDISIDNGNEDLAEILQ |
| NIL1 | GKKLLEAARAGQDDEVRILMANGADVNA/STICGATPLHLAANSGHLEIVEVLLKNGADVNA/SGNIGVTPLHLAAFMGHLEIVEVLLKYGADVNA/TMHYGITPLHLAAFWGHLEIVEVLLKNGADVNA/QNHSGKTAFDISIDNGNEDLAEILQ |
| NIL3 | GKKLLEAARAGQDDEVRILMANGADVNA/STWWGSTPLHLAAHYGHLEIVEVLLKNGADVNA/RQLCGSTPLHLAAVGGHLEIVEVLLKHGADVNA/TGNVGITPLHLAASWGHLEIVEVLLKYGTDVNA/QTRRGRTAFDISIDNGNEDLAEILQ |
| NIL7 | GKKLLEAARAGQDDEVRILMANGADVNA/FAESGRTPLHLAAVFGHLEIVEVLLKYGADVNA/HKFFGFTPLHLAASEGHLEIVEVLLKNGADVNA/TTILGSTPLHLAALRGHLEIVEVLLKHGTDVNA/QCDNGATAFDISIDNGNEDLAEILQ |
| NIL10 | GKKLLEAARAGQDDEVRILMANGADVNA/VNDCGITPLHLAARLGHLEIVEVLLKNGADVNA/SMCSGLTPLHLAAWGGHLEIVEVLLKDGADVNA/FWWMGITPLHLAAIRGHLEIVEVLLKHGADVNA/QTIWGFTAFDISIDNGNEDLAEILQ |

**Supplemental Figure S1B** – ELISA of 2 µM purified DARPins added to wells with or without IL-10. The ELISA plate was coated either with streptavidin or streptavidin plus 20 nM biotinylated IL-10. After blocking, 100 µL of each DARPin (final 2 µM) was added to each well.

**Supplemental Figure S2** – TGF-β1-binding DARPin sequences

| Motif | NCAP / AR1 / AR2 / AR3 / CCAP / |
| --- | --- |
| Clone | GKKLLEAARAGQDDEVRILMANGADVNA/XXXXGXTPLHLAAXXGHLEIVEVLLKXGADVNA/XXXXGXTPLHLAAXXGHLEIVEVLLKXGADVNA/XXXXGXTPLHLAAXXGHLEIVEVLLKXGADVNA/QXXXGXTAFDISIDNGNEDLAEILQ |
| 3A2 | GKKLLEAARAGQDDEVRILMANGADVNA/HSSFGCTPLHLAAGPGHLEIVEVLLKNGADVNA/FSRFGNTPLHLAAGRGHLEIVEVSLKHGADVNA/ADIRGATPLHLAALAGHLEIVEVLLKNGADVNA/QNSTGATAFDISIDNGNEDLAEILQ |
| 3B12 | GKKLLEAARAGQDDEVRILMANGADVNA/CCGRGFTPLHLAAMMGHLEIVEVLLKNGADVNA/EDGFGNTPLHLAAMGGHLEIVEVLLKNGADVNA/HDSLGTTPLHLAASVGHLEIVEVLLKHGADVNA/QWLPGLTAFDISIDNGNEDLAEILQ |
| 3C2 | GKKLLEAARAGQDDEVRILMANGADVNA/RTIIGCTPLHLAASYGHLEIVEVLLKHGADVNA/ALDRGLTPLHLAAGNGHLEIVEVLLKNGADVNA/HLLCGATPLHLAALTGHLEIVEVLLKHGADANA/QRNFGSTAFDISIDNGNEDLAEILQ |
| 3D11 | GKKLLEAARAGQDDEVRILMANGADVNA/RDLKGNTPLHLAACFGHLEIVEVLLKHGADVNA/WMLDGSTPLHLAACRGHLEIVEVLLKYGADVNA/PDNMGQTPLHLAAIFGHLEIVEVLLKDGADVNA/QINLGKTAFDISIDNGNEDLAEILQ |
| 3F8 | GKKLLEAARAGQDDEVRILMANGADVNA/VTCMGYTPLHLAACSGHLEIVEVLLKDGADVNA/SWYLGMTPLHLAAPHGHLEIVEVLLKYGADVNA/QLITGYTPLHLAAPMGHLEIVEVLLKNGADVNA/QKYSGLTAFDISIDNGNEDLAEILQ |
| 3G7 | GKKLLEAARAGQDDEVRILMANGADVNA/TTINGCTPLHLAAMGGHLEIVEVLLKNGADVNA/GMYEGSTPLHLAAYSGHLEIVEVLLKHGADVNA/PLTCGSTPLHLAALHGHLEIVEVLLKNGADVIA/QPDQGLTAFDISIDNGNEDLAEILQ |
| 3H2 | GKKLLEAARAGQDDEVRILMANGADVNA/KGWCGVTPLHLAARLGHLEIVEVLLKHGADVNA/RVECGATPLHLAASRGHLEIVEVLLKHGADVNA/NSHVGRTPLHLAALAGHLEIVEVLLKDGADVNA/QRKSGSTAFDISIDNGNEDLAEILQ |
| 3H7 | GKKLLEAARAGQDDEVRILMANGADVNA/AHACGFTPLHLAAYGGHLEIVEVLLKHGADVNA/LAKWGSTPLHLAAALGHLEIVEVLLKNGADVNA/TDPLGTTPLHLAAIIGHLEIVEVLLKYGADVNA/QCRLGRTAFDISIDNGNEDLAEILQ |
| 3H9 | GKKLLEAARAGQDDEVRILMANGADVNA/TDDRGWTPLHLAAWNGHLEIVEVLLKHGADVNA/GLTKGGTPLHLAAGGGHLEIVEVLLKHGADVNA/NLLNGPTPLRLAANVGHLEIVEVLLKNGADVNA/QMFTGITAFDISIDNGNEDLAEILQ |

**Supplemental Figure S3.** ELISA of 2 µM purified DARPins added to wells with or without TGFβ. The ELISA plate was coated either with streptavidin or streptavidin plus 10 nM biotinylated TGFβ. After blocking, 100 µL of each DARPin (final 2 µM) was added to each well.

**Supplemental Figure S4** – Engineering of DARPin A4. Phage ELISA of three rounds of phage panning against PD-L1. Phage from each round of panning were added to a MaxiSorp ELISA plate coated with 100 µL of 20 nM biotinylated PD-L1. Bound phage were detected with anti-M13 antibody conjugated to HRP.

**Supplemental Figure S5** – PD-L1-binding DARPin sequences

| Motif | NCAP / AR1 / AR2 / AR3 / CCAP / |
| --- | --- |
| A4 Parent | GKKLLEAARAGQDDEVRILMANGADVNA/SDSAGLTPLHLAARPGHLEIVEVLLKNGADVNA/PDLWGRTPLHLAAETGHLEIVEVLLKDGADVNA/DDAPGMTPLHLAAQGGHLEIVEVLLKYGADVNA/QDKFGKTAFDISIDNGNEDLAEILQ |
| 1F8 | GKKLLEAARAGQDDEVRILMANGADVNA/SDSAGLTPLHLAARPGHLEIVEVLLKNGADVNA/PDLWGRTPLHLAAETSHLEVVEVLLKDGADVNA/DDAPGMTPLHLAALGGHLEIVEVLLKYGADVNA/QDKYGKTAFDISIDNGNEDLAEILQ |
| 1D10 | GKKLLEAARAGQDDEVRILMANGADVNA/SDSAGLTPLHLAARPGHLEIVEVLLKNGADVNA/TDLWGRTPLHLAAESSHLEIVEVLLKDGADVNA/GDAPGMTPLHLAAQGGHLEIVEVLLKHGTDVNA/QDKFGKTAFDISIDYCNEDLAEILQ |
| 1B11 | GKKLLEAARAGHDDEVRILMANGADVNA/SDSAGLTPLHLAARPGHLEIVEVLLKNGADVNA/PDLWGRTPLHLAAETSHLEIVEVLLKDGADVNA/EDAPGMTPLHLAALGGHLEIVEVLLKYGADVNA/QDKFGKTAFDISIDNGNEDLAEILQ |
| 1E8 | GKKLLEAARAGQDDEARILMANGADVNA/SDSAGLTPLHLAARHGHLEIVEVLLKNGADVNA/PDLWGRTPLHLAAETSHLEIVEVLLKDGADVNA/DDAPGMTPLHLAAQGGHLEIVEVLLKYGADDNA/QDEFGMTAFDISIDDGNEDLAEILQ |
| 1E9 | GKKLLEAARAGQDDEVRILMANGADVNA/SDSAGLTPLHLAARPGHLEIVEVLLRNGADVNA/TDLWGRTPLHLAAETSHLEIVEVLLKDGADVNA/DDAPGMTPLHLAAQGGHLEIVEVLLKYGADVNA/QDKFGKTAFDISIDNGNEDLAEILQ |
| 1D6 | GKKLLEAARAGQDDEVRILMANGADVNA/SDSVGLTPLHLAARPGHLEIVEVLLKNGADVNA/PDLWGRTPLHLAAETTHLEIVEVLLKDGADVNA/DDAPGMTPLHLAAQGGHLEIAEVLLKYGADVNA/QDKFGKTAFDISIDNGNEDLAEILQ |
| 1E3 | GKKLLEAARAGQDDEVRILMANGADVNA/SDSAGSTPLHLAARPGHLEIVEVLLKYGAGVNA/PDLWGRTPLHLAAETSHLEIVEVLLKDGADVNA/DDAPGMTPLHLAAQGGHLEIVEVLLRYGADVNA/QDKFGKTAYDISIDNGNEDLAEILQ |
| 1A4 | GKKLLEAARAGQDDEVRILMANGADVNA/SDPAGLTPLHLAARPGHLEIVEVLLKNGADVNA/PDLWGRTPLHLAAETSHLEIVEVLLKDGADVNA/DDAPGMTPLHLAAQGGHLEIVEVLLKYGADVNA/QDKFGKTAFDISIDNGNEDLAEILQ |

**Supplemental Figure S6.** BLI binding kinetics of DARPin 1D10 to biotinylated human PD-L1. Color curves represent measured binding response. The BLI experiment was conducted using the BLItz instrument (ForteBio) with streptavidin-coated (SA) biosensors (Sartorius). PBS supplemented with 0.1% BSA and 0.02% Tween-20 was used for all sample dilution, sensor equilibration and baseline steps. The sensor was loaded with 150 nM of biotinylated PD-L1. Data was collected and analyzed using BLItzPro1.3 software.

**Supplemental Figure S7** – Nucleotide sequence of click display library construct.

atacgaaattaatacgactcactatagggagaccacaacggtttccctctagaaataattttgtttaactttaagaaggaggatatatccatgggcagcagccaccatcaccaccatcatcatcacagcagcggcggagaacaaaaactgatcagcgaagaggatctgGGATCCGATCTGGGTAAGAAACTTTTGGAGGCGGCCCGTGCCGGGCAAGACGACGAAGTGCGCATTTTGATGGCTAACGGGGCCGACGTTAACGCCNNKNNKNNKNNKGGTNNKACGCCTCTTCACTTGGCCGCANNKNNKGGCCACTTGGAGATCGTCGAAGTCTTACTGAAANNKGGCGCTGACGTGAACGCCNNKNNKNNKNNKGGANNKACGCCACTTCATTTAGCAGCCNNKNNKGGACACTTGGAAATTGTTGAGGTGTTACTTAAANNKGGGGCGGATGTTAATGCGNNKNNKNNKNNKGGGNNKACACCACTGCACCTGGCGGCANNKNNKGGACATTTAGAAATTGTGGAGGTGTTGTTAAAANNKGGTGCAGATGTCAATGCACAGNNKNNKNNKGGGNNKACGGCGTTCGATATCtccattgataacgggaacgaggatttagcagaaatccttcaaggtaccggaggtcctactctcctcaaggaaggcaaaaggacggggggcggcgtggaataactagcataaccccttggggcctctaaacgggtcttgaggggttttttg

T7 Promoter

Ribosome Binding Site

8X His tag

Myc tag

DARPin sequence (NNK randomized positions)

ML annealing region

T7 terminator
